# A Case of Carnitine Palmitoyltransferase II Deficiency in Bahrain With a Novel Mutation

**DOI:** 10.7759/cureus.26043

**Published:** 2022-06-17

**Authors:** Zahra Alsahlawi, Zainab Fadhul, Ali Mahmood, Ali Mohamed, Mohamed Khalil, Emtithal Aljishi

**Affiliations:** 1 Department of Pediatrics, Government Hospitals, Salmaniya Medical Complex, Manama, BHR; 2 Department of Pediatrics, Arabian Gulf University, Manama, BHR; 3 Department of Pediatrics, Royal College of Surgeons in Ireland, Medical University of Bahrain, Busaiteen, BHR; 4 Department of Pediatrics, King Hamad University Hospital, Busaiteen, BHR

**Keywords:** metabolic disease, carnitine palmitoyltransferase ii deficiency, pediatric genetic disease, novel mutation, l-carnitine, preimplantation genetic diagnosis

## Abstract

Carnitine palmitoyltransferase II (CPT II) deficiency is a rare genetic metabolic disorder. Three forms of the disease have been described: the lethal neonatal form, the severe infantile hepatocardiomuscular form, and the myopathic form. We report a case of the infantile form of CPT II deficiency with a novel mutation. Our patient is a seven-year-old Bahraini male who was investigated by the pediatric metabolic team following the sudden death of his twin sister in infancy. A fatty acid metabolic disorder was suspected based on his echocardiogram and tandem mass spectrometry (TMS) findings. Genetic analysis was initially inconclusive. Nonetheless, he was started on a fat-free diet, L-carnitine, and medium-chain triglycerides (MCT). At nearly two years of age, the patient had a metabolic crisis precipitated by a viral illness. TMS during this time was consistent with CPT II deficiency. Sanger sequencing then identified the presence of the variant c.161T>G (p.ille54Ser) in a homozygous state, confirming the diagnosis. Although this mutation has not been reported before in previous literature concerning CPT II deficiency, it is extremely likely that this mutation is pathogenic. Although the initial work-up of the patient was inconclusive, our clinical judgment was paramount in managing the patient.

## Introduction

Fatty acid oxidation allows cells to produce energy sources needed for metabolism in the absence of glucose [1,2]. This process occurs within the mitochondrial matrix and is contingent on the utilization of long-chain fatty acids (LCFA) present within the cytosolic compartment of cells. Given the impermeability of the mitochondrial membranes to LCFA, a shuttle is required to facilitate their transport from the cytosol into the mitochondria. Acetyl-CoA synthase, carnitine palmitoyltransferases (CPT I and II), and carnitine-acylcarnitine translocase are the key enzymes involved in this shuttle [1].

CPT II deficiency (OMIM 600650) is an autosomal recessive disorder where LCFA transport into the mitochondria is compromised, resulting in impairment of fatty acid oxidation and a wide array of clinical manifestations [3]. The first case was reported in 1973 in the form of myoglobinuria in adults [4]. To date, three forms of CPT II deficiency have been reported in the literature: the lethal neonatal form, the severe infantile hepatocardiomuscular form, and the myopathic form [5]. The least common, the lethal neonatal form, is invariably fatal and usually presents within the first few days of life with malignant arrhythmias, seizures, liver failure, dysmorphic features, and structural malformations [5,6]. The most common, the myopathic form, often but not always presents in adulthood with myoglobinuria and rhabdomyolysis secondary to triggers, such as fasting and exercise [5]. The infantile variety usually presents within the first year of life with cardiomyopathy, peripheral neuropathy, liver failure, and hypoketotic hypoglycemia [5,7]. These manifestations are typically precipitated by febrile illnesses and fasting. The literature has reported 28 cases of the infantile form to date [3]. We discuss the severe infantile form of CPT II deficiency and report a case of a seven-year-old patient with a novel mutation who presented to Salmaniya Medical Complex in Bahrain.

## Case presentation

The proband is a seven-year-old Bahraini male (twin B) born at 36 weeks of gestation as part of a set of twins to non-consanguineous healthy parents not known to have any genetic diseases. Due to a failure to conceive after three years of attempting to do so, the parents underwent in-vitro fertilization in 2014, which resulted in a dichorionic diamniotic twin pregnancy. No perinatal complications were reported. The twins were delivered at 36 weeks through a lower segment Cesarean section due to breech presentation of the female (twin A). Twin A and twin B weighed 1.6 kg and 1.7 kg, respectively. Both were admitted to the neonatal intensive care unit (NICU) for weight gain as per hospital protocol and were discharged in good health after two weeks.

At 10 weeks of age, the female twin presented to the pediatric emergency room with a one-day history of hypoactivity and forceful, continuous vomiting of milk. She was critically ill with tachycardia and hypotension on presentation necessitating admission to the pediatric intensive care unit (PICU). Routine blood, imaging investigations, and an echocardiogram were quickly done. She was found to have hypoglycemia, cardiomyopathy, and appeared septic. This raised suspicion of an inborn error of metabolism. She died on the same day in hospital due to severe cardiomyopathy. No specific diagnosis was found. The family was advised to thoroughly investigate her twin brother to prevent a similar outcome as his sister.

Within days of his sister's death, our patient underwent a screening echocardiogram that revealed mild left ventricular and septal hypertrophy (left ventricular ejection fraction of 48%, separation fraction of 23%, left ventricular posterior wall in systole of 6 mm, interventricular septum in systole of 7.9 mm). The acylcarnitine profile revealed very low free carnitine levels at 0.68 uM (reference range: 6-72 uM). This severe carnitine deficiency was consistent with a diagnosis of systemic carnitine deficiency. As such, a molecular genetic sequencing analysis of the *SLC22A5 (OCTN2)* gene was conducted at three months of age for diagnostic confirmation; however, it was negative. Despite those findings, our clinical suspicion of a fatty acid oxidation defect remained. Therefore, the patient was started on L-carnitine and medium-chain triglyceride (MCT) oil with diet modification for a suspected fatty acid oxidation disorder. The L-carnitine dosage was adjusted according to the results of the routinely performed TMS and his weight.

The patient remained stable on the treatment with occasional presentations of mild upper respiratory tract infections that were managed conservatively with no exacerbations of his clinical status. By the age of 12 months, the patient experienced a metabolic crisis (glucose of 1.7 mmol/L [reference range 3.5-5.5 mmol/L], ammonia of 42 umol/L [reference range 11-35 umol/L], creatine kinase of 2423 U/L [reference range <228 U/L]). His lactate was normal at 0.7 mmol/L (reference range 0.4-2 mmol/L). The patient was admitted and managed in a different hospital with no TMS done at that time. We discussed the case with the pediatric team at that hospital and provided our recommendations for treatment. We also advised giving the parents a TMS filter paper to present to any hospital at the time of another metabolic crisis.

Nine months later, at the age of 21 months, our patient was admitted to our hospital with fever, vomiting, and reduced feeding. He was clearly in another metabolic crisis, as indicated by his laboratory results which showed hypoglycemia (glucose of 1.4 mmol/L [reference range 3.5-5.5 mmol/L]), leukocytosis (white blood cell count of 12.7 [reference range 3.6-9.6]), hyperammonemia (51 umol/L [reference range 11-35 umol/L]), and high lactate dehydrogenase (400 U/L [reference range 100-300 U/L]). His creatinine kinase was normal (107 U/L [reference range <228 U/L]) this time. The parents handed the TMS filter paper, and analysis of acylcarnitine was instantly performed. It showed a high C16 value at 10.48 and a high C18:1 value at 10.80 (Table 1). For this reason, a second molecular genetic testing through Sanger sequencing of the CPT II gene was performed, which resulted in the identification of the variant c.161T>G (p.ille54Ser) in a homozygous state, confirming the diagnosis. Both parents were tested and found to be heterozygote for the mutation. Following confirmation of his diagnosis, our patient did not experience any illnesses requiring admission for four years. He presented several times to the emergency department for fever and vomiting, but no exacerbations of his clinical condition required admission, and he was managed successfully by his parents at home.

**Table 1:** Patient's tandem mass spectrometry results during the metabolic crisis at the age of 21 months

Analytes	Calculated value	Cutoff value*
Acylcarnitines	High, C12 = 0.70	0.35
	High, C14 = 1.24	0.60
	High, C16 = 10.48	7.00
	High, C18 = 3.49	2.00
	High, C16:1 = 0.97	0.60
	High, C18:1 = 10.80	2.50
Organic acids	Normal	
Amino acids	Normal	

At the age of six years, our patient tested positive for the novel coronavirus (COVID-19). This precipitated an attack of metabolic crisis with creatine kinase (CK) level of 726 U/L (reference range <228 U/L) on presentation and mandated admission. He remained in hospital for a week for supportive treatment. His laboratory results improved throughout the hospital course, and he was discharged home in good clinical condition with no further complications.

The patient has been managed in a multidisciplinary team approach in our hospital since he was a toddler. He has regular follow-up appointments with the metabolic team as well as the hospital's dietician and the pediatric cardiologist. Overall, he has met all appropriate developmental milestones for his age and is performing well in school. From a cardiac point of view, he has undergone eight echocardiograms to date. They all remarked a good overall systolic function, except for his last echocardiogram, which showed progressive concentric hypertrophy of the myocardium despite good overall function. This was found to be secondary to non-compliance to his fat-free diet in his last visit to the metabolic clinic.

In 2019, the parents expressed interest in having another child. We advised them to perform a pre-implantation genetic diagnosis (PGD) before conceiving in light of their experience with their older children. PGD was done, and the metabolic team was involved in planning and supporting the family throughout their pregnancy. In October 2020, the family welcomed a new baby who is a carrier of the mutation we describe in this paper. Our patient's sibling is now a two-year-old girl who is in good health.

## Discussion

Our patient is a confirmed case of CPT II deficiency, a rare autosomal recessive long-chain fatty acid oxidation disorder. Three types of CPT II deficiency were described in the literature: the lethal neonatal form, the severe infantile hepatocardiomuscular form, and the mildest and most common myopathic form. Our patient and his deceased sister fit the clinical diagnostic criteria for the infantile form of CPT II deficiency [5]. In our case, these symptoms included presentation during the first year of life with metabolic crisis and cardiomyopathy [5,8], leading to the death of twin A.

The severe infantile form has been reported in 28 families around the world [3]. In a review of the literature, we identified 16 different cases across 14 families [7-19]. Of those reported cases, three originated from the Middle East and North Africa (MENA) area [9,15,17]. Our report describes the first case in Bahrain and the Gulf Cooperation Council region.

An array of clinical manifestations can be described in the severe infantile form of CPT II deficiency. Typical presentations involve the liver in the form of acute liver failure and hypoglycemia precipitating seizures and coma, as well as the heart in the form of cardiomyopathies and arrhythmias [5]. Paroxysmal heartbeat disorders are thought to be the cause of sudden infant death during the first year of life [8-10,12,14]. This could explain the sudden deterioration in our patient's twin sister's health and her death at the age of 10 weeks of life. It is also worth mentioning that this presentation in the twin sister led to our suspicion of a metabolic disorder in our patient, and hence, the quick action in undergoing numerous investigations, including an echocardiogram, TMS, and genetic testing.

Metabolic crises are also a typical presentation of the infantile form of CPT II deficiency. They are usually precipitated by febrile illnesses or fasting. However, they can also occur independently from these causes [5]. In metabolic crises, physicians should pay particular attention to laboratory results that reveal metabolic acidemia, hyperammonemia, and hypoglycemia. Our patient had a total of six admissions to our hospital for metabolic crises in the first two years of his life. They were all precipitated by upper respiratory tract infections or gastritis. He also had one attack requiring admission and management in a different hospital. We provided our recommendations to the treating physicians at that hospital and handed the parents a TMS paper test to perform at the time of another crisis. His sixth and last admission to our hospital was at the age of 21 months and led to his diagnosis of CPT II deficiency. The parents handed the TMS paper as instructed, and the TMS of acylcarnitines showed high C16 and C18:1, confirming a diagnosis of CPT II deficiency [20]. Afterward, a second genetic test was sent and identified the variant *c.161T>G (p.ille54Ser)* in a homozygous state. The amino acid isoleucine at position 54 affects a highly conserved amino acid and is located in the acyltransferase ChoActase protein domain, which suggests that this mutation is pathogenic. Both parents tested as heterozygous for the same mutation, excluding allelic loss. To the best of our knowledge, this change was not reported before.

Since its introduction, the acylcarnitine profile by TMS has provided an efficient means to distinguish between the different fatty acid oxidation disorders. Characteristic profiles have been linked to specific diseases with similar clinical manifestations [20]. CPT II deficiency classically results in the elevation of serum long-chain acylcarnitines: palmitoylcarnitine (C16) and oleoylcarnitine (C18:1) [20]. This was observed in our patient at the time of crisis. It is important to note, however, that while TMS usually points towards the underlying fatty acid oxidation disorder, physicians might encounter atypical results, especially between crises [20] which was observed in our patient whose initial TMS was suggestive of systemic or primary carnitine deficiency and subsequent additional TMS analyses between attacks were normal.

Atypical presentations of CPT II deficiency affecting the central nervous system were also described in the literature [5]. However, this has not been noted in our patient, who is developmentally normal with no neurological manifestations.

Since our suspicion of a fatty acid oxidation disorder was raised in the infantile period, our patient was treated with L-carnitine, MCT oil, and a fat-free diet. While the use of L-carnitine is controversial because it is hypothesized that it may lead to the paradoxical effect of mitochondrial depletion of acyl-CoA [21], we noticed that clinically our patient was well controlled from the age of two after diagnosis with no further metabolic crises for over four years. We constantly check the level of free total carnitine in the blood to adjust the dose of L-carnitine in follow-up appointments, and our patient has not suffered any side effects from this treatment.

At the age of six years, however, our patient contracted COVID-19, which led to a metabolic crisis. He was admitted to the hospital for observation and supportive treatment and was discharged home in good condition. It is worth mentioning that the novel SARS-CoV-2 has been reported to precipitate metabolic crises in patients with inborn errors of metabolism. An observational study of 11 patients with different types of inborn error of metabolism who contracted SARS-CoV-2 concluded that the virus could precipitate a serious metabolic crisis in patients known to have an inborn error of metabolism. Of note, the metabolic disease itself would not necessarily affect the natural course of the viral illness [22].

Genetic counseling was provided to this family prior to the conception of another child. We explained to the parents that they were heterozygote for the mutation and advised PGD before the pregnancy. The family then underwent IVF and welcomed a healthy asymptomatic carrier of CPT II deficiency in October 2020. We are the first to describe the utilization of PGD for a family with CPT II deficiency in the literature.

## Conclusions

This case highlights typical features of the infantile form of CPT II deficiency. Negative work-up may be seen initially, but clinical judgment is paramount in approaching these patients as initiating early treatment can be lifesaving and significantly improves the quality of life. In acute illnesses, physicians should pay attention to laboratory results that can show metabolic acidemia, hyperammonemia, and hypoglycemia. Although controversial, L-carnitine supplementation, together with regular and careful monitoring, appears to be beneficial. Genetic counseling should be offered for families with metabolic illnesses to help in family planning.
